# Recent advances in the management of gastric adenocarcinoma patients

**DOI:** 10.12688/f1000research.15133.1

**Published:** 2018-08-30

**Authors:** Kazuto Harada, Anthony Lopez, Namita Shanbhag, Brian Badgwell, Hideo Baba, Jaffer Ajani

**Affiliations:** 1 Department of Gastrointestinal Medical Oncology, University of Texas M.D. Anderson Cancer Center, 1515 Holcombe Blvd., Houston, TX 77030, USA; 2 Department of Gastroenterological Surgery, Graduate School of Medical Science, Kumamoto University, 1-1-1 Honjo, Kumamoto 860-8556, Japan; 3 Department of Gastroenterology and Hepatology and Inserm U954, Nancy University Hospital, Lorraine University, 5 allée du Morvan, 54511 Vandoeuvre-lès-Nancy, France; 4 Department of Surgical Oncology, University of Texas M.D. Anderson Cancer Center, 1515 Holcombe Blvd., Houston, TX 77030, USA

**Keywords:** Gastric adenocarcinoma, preoperative therapy, targeted therapy

## Abstract

Gastric adenocarcinoma (GAC) is one of the most aggressive malignancies and has a dismal prognosis. Therefore, multimodality therapies to include surgery, chemotherapy, targeted therapy, immunotherapy, and radiation therapy are needed to provide advantage. For locally advanced GAC (>cT1B), the emerging strategies have included preoperative chemotherapy, postoperative adjuvant chemotherapy, and (occasionally) postoperative chemoradiation in various regions. Several novel therapies have been assessed in clinical trials, but only trastuzumab and ramucirumab (alone and in combination with paclitaxel) have shown overall survival advantage. Pembrolizumab has been approved by the US Food and Drug Administration on the basis of response rate only for patients with microsatellite instability (MSI-H) or if PD-L1 expression is positive (≥1% labeling index in tumor/immune cells in the presence of at least 100 tumor cells in the specimen). Nivolumab has been approved in Japan on the basis of a randomized trial showing significant survival advantage for patients who received nivolumab compared with placebo in the third or later lines of therapy. The cure rate of patients with localized GAC in the West is only about 40% and that for metastatic cancer is very poor (only 2–3%). At this stage, much more target discovery is needed through molecular profiling. Personalized therapy of patients with GAC remains a challenge.

## Introduction

Gastric adenocarcinoma (GAC) is considered the fifth most common cancer in the world (1,313,000 cases) and the third leading cause of cancer death globally (819,000 deaths) ^1^. Its incidence varies according to the region: high incidence in East Asia and South America but low incidence in the West ^1^. In East Asia, especially Japan and Korea, the incidence of distal GAC is high, whereas proximal GAC has a higher incidence in the West ^2^. GAC located in the cardia or the gastroesophageal junction (or both) has dramatically increased in incidence in the USA ^3^. This trend of proximal migration of GAC is also being observed in Asia and South America along with Europe. Based on American Joint Committee on Cancer 8 (AJCC 8), gastroesophageal junction adenocarcinoma that has its epicenter in the proximal 2 to 5 cm of the stomach (Siewert type III) should be staged and treated as GAC. For locally advanced GAC, various strategies have been developed in different regions of the world and these have evolved on the basis of practice preferences and types of clinical trials performed. For example, preoperative chemotherapy is favored in the European Union and the USA, whereas postoperative adjuvant chemotherapy is preferred in Asia. Postoperative chemoradiation has a diminishing role while the quality of surgery appears to be improving. Adjunctive therapy (preoperative or postoperative) seems to increase the cure rate by about 10%. Multidisciplinary evaluation is essential to improve patient outcomes and for the initial treatment decision process.

For metastatic GAC, the standard-of-care therapies have a limited impact on patient outcome. The median survival of patients with advanced GAC is less than 12 months. Only a limited number of therapies are approved, and many of these therapies are done empirically. Here, we summarize recent advances in the management of GAC.

## Molecular features of gastric adenocarcinoma

Two groups—The Cancer Genome Atlas (TCGA) and Asian Cancer Research Group (ACRG)—have reported multiplatform sequencing of primary GACs (TCGA analysis being more comprehensive than ACRG) and, out of these efforts, four genotypes of GAC have emerged ^4, 5^. TCGA data were more comprehensive and basically GACs have been divided into those with microsatellite instability (MSI), chromosomal instability (CIN), genome stability (GS), and Epstein–Barr virus (EBV) association ^5^. The MSI cohort represented about 22% and was more frequent in the distal GAC than the proximal GAC. In contrast, CIN is more frequent in the proximal GAC. Compared with other gastrointestinal adenocarcinomas, CIN in GAC tends to have focal region alterations ^6^. GS and EBV had frequencies of 20% and 9%, respectively.

Certain molecular subtypes are associated with shorter survival; for example, GS and CIN have poor prognosis ^4, 7^. The GS subtype of GAC is enriched in diffuse-type histology and is molecularly characterized by less mutation and overexpression of epithelial–mesenchymal transition-related genes ^4, 5^. On the other hand, the CIN subtype of GAC is enriched in intestinal histology and is molecularly characterized by *TP53* mutation and *RTK-RAS* activation/amplifications ^4, 5^. MSI subtyped GAC harbors numerous somatic mutations, leading to a large number of neoantigens that can activate T cells ^8^. Thus, GACs with MSI respond well to immune checkpoint blockade ^9^. However, the frequency of MSI-H and EBV-related GACs in the metastatic setting is low (<3%).

## Resectable gastric adenocarcinoma

### Primary resection

GAC with clinical T1N0 can be treated by either endoscopic therapy or surgery without any adjunctive therapy, while advanced localized GAC should undergo either preoperative therapy or surgery first followed by adjuvant chemotherapy. Adequate lymphadenectomy (D2 dissection) is desired with gastrectomy ^10^. If the depth of invasion suggests eusT1a, then endoscopic resection is preferred according to the Japanese guidelines ^10^. When surgery is performed first, postoperative adjuvant chemotherapy should be considered on the basis of the pathological stage or quality of surgery.

### Postoperative treatment

Postoperative adjuvant chemotherapy is the most common strategy in East Asia. The ACTS-GC trial, a phase III trial in Japan, showed that postoperative adjuvant therapy with S-1 for 12 months improved overall survival (OS) (5-year OS 72% versus 61%, hazard rate [HR] 0.67, 95% confidence interval [CI] 0.52–0.82) and relapse-free survival (RFS) (5-year RFS 65% versus 53%, HR 0.65, 95% CI 0.54–0.79) in GAC patients with stage II/III who underwent D2 gastrectomy ^11^. The CLASSIC trial, a phase III trial performed in South Korea, China, and Taiwan, showed that adjuvant capecitabine plus oxaliplatin combination given for 6 months after D2 gastrectomy improved OS (5-year OS 78% versus 69%, HR 0.66, 95% CI 0.51–0.85) and RFS (5-year RFS 68% versus 53%, HR 0.58, 95% CI 0.47–0.72) ^12^. These trials are the basis for recommending postoperative chemotherapy after optimal surgery. More intensive regimens have no advantage for postoperative chemotherapy ^13, 14^. Recently, the JACCRO GC-07 trial suggests that S-1 plus docetaxel has significant advantage over S-1 alone and it has become the standard of care ^15^.

Efficacy of postoperative chemoradiation was shown in the INT-0116 trial; compared with the surgery-alone group, the postoperative chemoradiotherapy group had longer OS (median OS 27 versus 36 months, HR 1.35, 95% CI 1.09–1.66) and RFS (median RFS 19 versus 30 months, HR 1.52, 95% CI 1.23–1.86) ^16, 17^. However, the quality of surgery was suboptimum in patients enrolled in this trial; D0, D1, and D2 lymph node dissection rates were 54%, 36%, and 10%, respectively. Importantly, the ARTIST and CRITICS trials demonstrated the lack of efficacy of postoperative chemoradiation after D2 or D1+ nodal dissection ^18, 19^. Therefore, postoperative chemoradiation is not useful if optimal or near-optimal surgery is performed.

### Preoperative treatment

The MAGIC trial provided evidence that perioperative chemotherapy (three preoperative and three postoperative cycles of epirubicin, cisplatin, and fluorouracil [ECF]) for resectable GAC could improve cure rates (5-year OS 23% versus 36%, HR 0.75, 95% CI 0.60–0.93) ^20^. However, a follow-up analysis of the MAGIC results has not yet been presented. The results overall were suboptimal. Moreover, epirubicin is not necessary ^21^. The FNCLCC/FFCD trial showed that surgery plus preoperative cisplatin and fluorouracil (FP) improved OS compared with surgery alone (5-year OS 24% versus 38%, HR 0.69, 95% CI 0.50–0.95) ^22^. Recently, the MRC-OEO5 trial compared two cycles of FP and four cycles of ECF as perioperative chemotherapy and showed that the two regimens had similar OS (3-year rate 42% versus 39%, HR 0.92, 95% CI 0.79–1.08) ^23^. These results suggest that the addition of epirubicin and longer duration of chemotherapy do not provide any advantage.

The FLOT4 trial compared perioperative (predominantly preoperative) chemotherapy with docetaxel, oxaliplatin, and fluorouracil/leucovorin (FLOT) and ECF/ECX ^24, 25^. A total of 716 patients were randomly assigned to the ECF/ECX group (n = 360) or the FLOT group (n = 356). FLOT improved median RFS (30 versus 18 months, HR 0.75, *p* = 0.001) and median OS (50 versus 35 months, HR 0.77, *p* = 0.012) compared with ECF/ECX. A total of 50% of patients in the FLOT group completed the planned postoperative treatments, while 37% of patients in ECF/ECX completed them. Perioperative complications were similar across the two groups and unacceptably high ^24, 25^. Grade 3–4 diarrhea, infections, sensory disorder, and neutropenia were more frequent in the FLOT group, while vomiting, nausea, thromboembolism, and anemia were more frequent in the ECF/ECX group. FLOT should not be recommended to every patient. FLOT might be more suitable for only very fit patients with GAC rather than patients with esophageal or gastroesophageal junction adenocarcinoma where chemoradiation followed by surgery is the preferred strategy. FLOT resulted in 90-day mortality of 5% and grade 3–4 diarrhea (10%) and infections (18%). The follow-up in this trial is also short, and it is likely that, with further follow-up, the differences between the two regimens will narrow. If the 5-year OS rate of FLOT is about 40%, then it would not be a major advance.

The efficacy of targeted therapy for perioperative treatment was assessed. The ST03 trial evaluated whether adding bevacizumab to perioperative chemotherapy could improve survival ^26^. A total of 1,063 patients were randomly assigned to receive chemotherapy alone (n = 533) or chemotherapy plus bevacizumab (n = 530), and prognosis was similar in the two groups (3-year OS 50.3% versus 48.1%, HR 1.08, 95% CI 0.91–1.29, *p* = 0.36) ^26^. Moreover, anastomotic leak was more frequent in the chemotherapy-plus-bevacizumab group ^26^. To date, there is no place for targeted therapy in the preoperative setting. PETRARCA/FLOT6 (ClinicalTrials.gov Identifier: NCT02581462) is evaluating the efficacy of adding herceptin/pertuzumab to perioperative chemotherapy for human epidermal growth factor receptor 2 (HER2)-positive GAC. RAMSES/FLOT7 (ClinicalTrials.gov Identifier: NCT02661971) is evaluating the efficacy of adding ramucirumab (VEGFR2 monoclonal antibody) for HER2-negative GAC.

For esophageal or gastroesophageal junction adenocarcinoma, the CROSS trial documented benefit of preoperative chemoradiation ^27^. For GAC, preoperative chemoradiation is an attractive but non-standard option. Several trials have evaluated the efficacy of preoperative chemoradiation ^28– 31^, and a retrospective study at the M.D. Anderson Cancer Center showed excellent prognosis of GAC patients who had preoperative chemoradiation ^32^. To date, phase III trials assessing the value of preoperative chemoradiation in GAC are ongoing. The TOPGEAR trial is assessing the efficacy of adding preoperative radiation to the MAGIC trial regimen ^33^. The CRITICS-II trial is comparing three arms: preoperative chemotherapy followed by surgery, preoperative chemotherapy and subsequent chemoradiation followed by surgery, and preoperative chemoradiation followed by surgery (ClinicalTrials.gov Identifier: NCT02931890). Result of these trials are expected.

## Standard treatment for metastatic patients

### Standard first-line therapy

The recommended first-line therapy for patients with HER2-negative GAC is a two-drug combination of oxaliplatin (preferred) or cisplatin plus 5-FU or capecitabine. For HER2-positive GAC, the ToGA study showed that trastuzumab should be added to the first-line cytotoxic therapy ^34^. Irinotecan or taxane should be considered when platinum-based chemotherapy cannot be tolerated in the first-line setting ^35– 37^. DCF (docetaxel, cisplatin, and 5-FU) provides marginal OS advantage but its toxicity is significant ^38, 39^. Therefore, a modified regimen has been evaluated ^40– 43^. Modified DCF is still one of the options in selected cases, but the routine use of a taxane in the first-line setting should be avoided. Also, ECF is not recommended for metastatic GAC ^44^.

### Standard second-/third-line therapy

In the second-line therapy setting, ramucirumab is the only molecular-targeted drug with a confirmed but marginal survival benefit (as a single agent) in a global phase III trial. The RAINBOW study showed that OS as a result of ramucirumab plus paclitaxel was significantly longer than in the placebo-plus-paclitaxel group (median OS 9.6 versus 7.4 months) and thus this regimen is the preferred choice for second-line therapy ^45^. Ramucirumab monotherapy is not recommended ^46, 47^. Docetaxel, irinotecan, and paclitaxel have significantly prolonged OS compared with best supportive care (BSC) ^48– 50^. However, the data on these molecules are based on a few small trials.

### Treatment for peritoneal metastatic gastric adenocarcinoma

There is no established therapy for peritoneal carcinomatosis, which is a common site of metastases in patients with advanced GAC. Systemic chemotherapy or BSC is often recommended ^28^. Recently, intraperitoneal chemotherapy or hyperthermic intraperitoneal chemoperfusion (HIPEC) has been assessed as a potential therapy for peritoneal metastases. PHOENIX-GC, a Japanese phase III trial, compared S-1 in combination with intravenous/intraperitoneal paclitaxel and S-1 in combination with intravenous cisplatin (standard therapy in Japan) ^51^. Primary results showed that there was no significant difference between the two groups (median OS 17.7 and 15.2 months), suggesting lack of superiority of the intraperitoneal chemotherapy ^51^. A subgroup appeared to benefit, but this would only be hypothesis generating ^52, 53^. Also, perioperative intraperitoneal chemotherapy is being assessed ^54, 55^. The CY-PHOENIX trial showed that intraperitoneal chemotherapy for CY-positive patients (no other site of metastasis) resulted in conversion to CY-negative in 36 patients (94.7%) and 84.2% of the 1-year OS rate ^55^. Neoadjuvant laparoscopic HIPEC (mitomycin C 30 mg and cisplatin 200 mg) was assessed in a phase II study, which showed that seven patients (37%) had negative peritoneal cytology after HIPEC ^56^.

## Molecularly targeted drug for metastatic gastric adenocarcinoma

### HER2

HER2 is the only molecularly targeted drug accepted in first-line therapy. The ToGA study showed that adding trastuzumab in chemotherapy for patients with HER2-positive GAC resulted in longer median OS (13.8 versus 11.1 months, HR 0.74, 95% CI 0.60–0.91) ^34^. However, the subsequent analysis of the ToGA study showed that all of these differences have decreased by 40%. HER2 status should be tested according to guidance in all candidates for HER2-targeted therapy ^57^. The randomized phase III GATSBY trial compared T-DM1, conjugated with trastuzumab and emtansine, and taxane in patients with HER2-positive GAC in a second-line setting ^58^. However, T-DM1 failed to show benefit in OS (median OS 7.9 versus 8.6 months, HR 1.15, 95% CI 0.87–1.51) ^58^.

TRIO-013/LOGiC, a randomized phase III trial, assessed lapatinib, a dual inhibitor of HER2 and epidermal growth factor receptor (EGFR) ^59^. However, the addition of lapatinib to chemotherapy did not show benefit in OS for patients with HER2-positive GAC in the first-line setting (median OS 12.2 versus 10.5 months, HR 0.91, 95% CI 0.73–1.12) ^59^. TyTAN, a randomized phase III study, also showed that lapatinib was not effective for patients with HER2-positive GAC in the second-line setting (median OS 11.0 versus 8.9 months, HR 0.84, 95% CI 0.64–1.11) ^60^.

### EGFR

EGFR-targeted therapies showed no benefit in any trials for GAC. Two trials—the EXPAND trial and the REAL3 study—failed to show a benefit from the addition of EGFR inhibitor to first-line chemotherapy ^61, 62^. To date, EGFR inhibitor is evaluated in selected patients who have EGFR overexpression with immunohistochemistry (IHC) 2+ or 3+ (ClinicalTrials.gov Identifier: NCT01813253).

### VEGFR

Ramucirumab is marginally effective for second-line therapy based on REGARD and performs somewhat better, as shown in the RAINBOW study ^45, 46^. The RAINFALL trial assessed whether adding ramucirumab to first-line standard chemotherapy could be effective ^63^. A total of 645 patients were randomly assigned to two groups—ramucirumab plus chemotherapy (n = 326) or placebo plus chemotherapy (n = 319)—and adding ramucirumab marginally prolonged progression-free survival (PFS) (median PFS 5.7 versus 5.4 months, HR 0.75, 95% CI 0.61–0.94) but not OS (median OS 11.2 versus 10.7 months, HR 0.96, 95% CI 0.80–1.16) ^63^. These results strongly suggest that ramucirumab should not be used as first-line therapy. Bevacizumab, in the first-line setting, was found to have no efficacy when added to chemotherapy in the AVAGAST study or perioperative chemotherapy in the STO3 study ^26, 64^.

### MET

The phase III RILOMET-1 trial compared ECX with and without MET-positive gastroesophageal adenocarcinoma, but this trial was stopped early because of a higher number of deaths in the rilotumumab group; the median OS was 8.8 months in the rilotumumab group (n = 304) compared with 10.7 months in the placebo group (n = 304) ^65^. The MET gastric trial assessed adding onartuzumab to FOLFOX in HER2-negative GAC but did not significantly benefit OS (median OS 11.0 versus 11.3 months, HR 0.82, 95% CI 0.59–1.15) ^66^. These results suggest that MET inhibitors are not particularly efficacious in most patients with MET-positive GAC.

### mTOR

The GRANITE-1 study compared everolimus, an inhibitor of mammalian target of rapamycin (mTOR), and placebo in patients with GAC previously treated with standard care ^67^. Unfortunately, everolimus showed no benefit for OS (median OS 5.9 versus 4.3 months, HR 0.90, 95% CI 0.75–1.08) ^67^. The PADPAC study assessed adding everolimus to paclitaxel in the second-line setting but did not show significant benefit; the median OS was 6.1 months in the everolimus group (n = 150) compared with 5.1 months in the placebo group (n = 150) ^68^.

### FGFR2

Fibroblast growth factor receptor (FGFR) inhibitor failed to show a benefit in a phase II trial, the SHINE study ^69^. GAC patients with FGFR2 amplification were randomly assigned to an AZD4547 (an FGFR inhibitor) group (n = 41) or a paclitaxel group (n = 30) and showed similar median PFS: 1.8 months in the AZD4547 group and 3.5 months in the paclitaxel group ^69^.

### PARP

Poly (ADP-ribose) polymerase (PARP) inhibitor is potentially useful in cancers with a deficiency in the repair of double-strand breaks ^70^. In patients with GAC, a phase II trial showed that lower/absent expression level of ATM, which has a key role in activating DNA damage response to double-strand breaks, seemed to benefit from PARP inhibitor ^71^. However, the phase III GOLD study did not show an advantage from the addition of olaparib ^72^. A total of 643 patients were assigned to the olaparib-plus-paclitaxel arm (n = 263) or the placebo-plus-paclitaxel arm (n = 262), and the median OS values in the olaparib and placebo groups were 8.8 and 6.9 months, respectively (not significant) ^72^. Even in the ATM-negative population, olaparib was not effective ^72^.

### Targeted therapies against stem cell pathway

Cancer stem cells are often resistant to therapy that is non-specific and thus can be targeted to overcome resistance ^73^. Thus far, few clinical trials have assessed the effect of inhibiting stemness-related pathways, such as the Hedgehog and signal transducer and activator of transcription 3 (STAT3) pathway. Vismodegib, an inhibitor of Hedgehog signal by binding smoothened (SMO), was assessed in a phase II randomized study, but adding vismodegib to FOLFOX did not prolong PFS (11.5 versus 9.3 months, *p* = 0.34) ^74^. Moreover, the BRIGHTER study showed that adding napabucasin, a STAT3 inhibitor, to paclitaxel did not prolong OS ^75^. The trial did not enrich patients by expression of stem cell markers in the tumor cells. It may be that selected patients should be assessed ^76^. STAT3 inhibitors may be better suited for immune modulation as well.

### Other targets

The isoform 2 of the tight junction molecule claudin-18 (CLDN18.2) is an important component of the tight cell junctions and overexpressed significantly in GAC ^77^. IMAB362, an inhibitor of CLDN18.2, was assessed in clinical trials. In a randomized phase II trial, IMAB362 plus epirubicin, oxaliplatin, and capecitabine (EOX) significantly prolonged PFS (median 5.7 versus 7.9 months, HR 0.5, 95% CI 0.35–0.78) ^78^. Moreover, the benefit of IMAB362 was more pronounced in the patients with CLDN18.2 high-expression (70% labeling index) GAC (PFS 6.1 versus 9.1 months, HR 0.46; OS 9.3 versus 16.6 months, HR 0.44) ^78^. A phase III trial is ongoing.

Matrix metalloproteinase 9 (MMP9) has a key role in extracellular matrix remodeling and angiogenesis, and its inhibition in combination with FOLFOX led to potential improvement of prognosis in a phase I study ^79^. A phase III trial assessing potential benefit of MMP9 inhibition has completed accrual (ClinicalTrials.gov Identifier: NCT02545504) ^80^.

## Immunotherapy

Immunotherapy checkpoint therapy has dramatically improved the prognosis of patients with metastatic melanoma or non-small cell lung cancer ^81, 82^, and the concept/approach has been adopted in many tumor types, including gastrointestinal malignancies. Cytotoxic T-lymphocyte associated protein 4 (CTLA-4) and programmed death protein 1 (PD-1) and its ligand (PD-L1) are the key proteins that have the capacity to inhibit the responses of T cells that can be tumor promoting. A few phase III trials assessing the efficacy of immune checkpoint inhibitors in gastrointestinal malignancies have been conducted in patients with advanced gastric or gastroesophageal junction cancer ^83^. After two or more prior therapy failures, 493 Asian patients were randomly assigned to receive nivolumab, a PD-1 antibody, at 3 mg/kg (n = 330) or placebo (n = 163) every 2 weeks. The median OS was 5.3 months in the nivolumab group versus 4.1 months in the control group ( *p* <0.0001). The median PFS was also slightly longer with nivolumab compared with placebo (HR 0.60, 95% CI 0.49–0.75). The safety profile was excellent; the grade 3 or 4 treatment-related adverse event rate was 10% in the nivolumab group. The combination of different immune checkpoint inhibitors may also be promising.

Ipilimumab is an anti-CTLA4 antibody, tested in the phase I/II study, in combination with nivolumab ^84^. Patients (n = 160) were randomly assigned to one of three groups: nivolumab alone (3 mg/kg), nivolumab plus ipilimumab (1 and 3 mg/kg, respectively), and nivolumab plus ipilimumab (3 and 1 mg/kg, respectively). Overall response rates (ORRs) were 12%, 24%, and 8% in the first, second, and third groups, respectively, and seemed better in the case of PD-L1 expression. Corresponding median OS values were 6.2, 6.9, and 4.8 months (not reached in PD-L1 ^+^ subgroups). Toxicity was increased with combination therapy (47% of grade 3 or 4 adverse events and treatment discontinuation due to toxicity in 20% of the cases in the second group). A phase III trial is ongoing.

Pembrolizumab is another anti-PD-1 antibody, currently tested alone or in combination in three different cohorts in the phase II KEYNOTE-059 study. In cohort 1, patients with advanced gastric and gastroesophageal junction cancer who were progressive after two or more chemotherapy lines received pembrolizumab monotherapy at 200 mg every 3 weeks ^85^. In total, 259 patients were included (52% in third-line and 48% in fourth-line or more). ORR, the primary endpoint, was 11.6%; interestingly, durable responses were observed in patients with PD-L1-positive cancer (15.5% in PD-L1 positive and 6.4% in PD-L1 negative). Based on these results, pembrolizumab has been approved by the US Food and Drug Administration (FDA) for patients with GAC positive for PD-L1 expression (≥1% labeling index in tumor/immune cells in the presence of at least 100 tumor cells in the specimen) to be used in the third (or later) line. Cohort 2 included treatment-naïve patients who received a combination of pembrolizumab (200 mg/3 weeks) and chemotherapy (cisplatin plus 5-FU or capecitabine). Preliminary results in 25 patients were promising, showing an ORR of 60% and a median PFS of 6.6 months ^86^. These results suggest that anti-PD1 antibodies can potentiate conventional cytotoxic chemotherapy in the first-line setting. The results from the phase III trial are expected (ClinicalTrials.gov Identifier: NCT02494583) ^87^.

Avelumab is an anti-PD-L1 antibody and was evaluated in the phase Ib JAVELIN trial as a first-line maintenance (group 1) or second-line (group 2) therapy ^88^. ORRs were 9.0% ** and 9.7% in groups 1 and 2, respectively. The corresponding ** median PFS values were 12.0 and 6.0 weeks. Two phase III trials ** are ongoing with avelumab in metastatic GAC ** or gastroesophageal junction adenocarcinoma. The JAVELIN Gastric 300 evaluated avelumab as a third-line treatment, and 371 patients were randomly assigned to receive avelumab plus BSC or chemotherapy (paclitaxel or irinotecan) plus BSC (ClinicalTrials.gov Identifier: NCT02625623). This trial failed to meet its primary outcome of superior survival, and detailed results are awaited. The JAVELIN Gastric 100 has been evaluating avelumab as a maintenance therapy (ClinicalTrials.gov Identifier: NCT02625610). This trial has completed accrual and results are awaited.

The modest efficacy and high cost of immune checkpoint inhibitors suggest that we need a reliable predictive/prognostic biomarker to select these agents. In studies described above, ORR was two to three times higher in the case of PD-L1 positivity in tumor cells. However, results are conflicting, and there is no consensus on the best way to assess PD-L1 status. MSI refers to the replicative error phenotype caused by mutations in the mismatch repair (MMR) system. Recently, Le *et al*. reported an ORR of 53% in 86 patients with MSI-H tumors, of whom 76% had gastrointestinal cancers ^89^. After a 2-year follow-up, 53% of the patients had not had tumor progression and 64% were still alive (medians not reached). In the KEYNOTE-059 study (cohort 1), the ORR was 57% in patients with MSI tumors compared with 9% in the case of MSS tumors ^85^. Based on five trials (KEYNOTE-016, KEYNOTE-164, KEYNOTE-012, KEYNOTE-028, and KEYNOTE-158), pembrolizumab has been approved by the FDA for MSI-H or deficient MMR solid tumors. In preliminary studies, a new generation of immunotherapy drugs is being evaluated for downregulating immunosuppressive pathways (for example, VISTA, an immunosuppressive molecule expressed on regulatory T cells, and indoleamine 2,3-dioxygenase (IDO), an enzyme leading to decreased tryptophan level which then suppresses T-cell proliferation) or stimulating immune tumor response (for example, inducible T-cell co-stimulator [ICOS] agonists) ^90^.

## Conclusions

In summary, in addition to high-quality surgery, preoperative or postoperative chemotherapy is recommended for localized advanced GAC, depending on local preferences ( Table 1). Preoperative chemoradiation is a non-standard option with potential and is being studied in phase III trials. Only trastuzumab and ramucirumab (in combination) are effective targeted drugs for GAC ( Table 2). PD-1 inhibitor is highly active against MSI-H GACs. Clearly, more basic research is needed to identify novel targets and drugs.

**Table 1.** Preoperative treatment trial for localized gastric adenocarcinoma.

Study	Number	Treatment	Survival	HR (95% CI)	*p* value	Reference
ACTS-GC	n = 529 n = 530	Surgery → S-1 Surgery	5-year OS: 72% 5-year OS: 61%	0.67 (0.54–0.82)	-	11
CLASSIC	n = 520 n = 515	Surgery → XP Surgery	5-year OS: 78% 5-year OS: 69%	0.66 (0.51–0.85)	0.0015	12
ITACA-S	n = 562 n = 538	Surgery → FOLFIRI → DP Surgery → 5-FU/LV	5-year OS: 51% 5-year OS: 51%	0.98 (0.82–1.18)	0.87	13
INT-0116	n = 281 n = 275	Surgery → 5-FU/45 Gy Surgery	Median OS: 36 months Median OS: 27 months	1.35 (1.09–1.66)	0.005	16
ARTIST	n = 228 n = 230	Surgery → XP Surgery → XP/45 Gy	3-year DFS: 74% 3-year DFS: 78%	-	0.86	18
CRITICS	n = 393 n = 395	ECC → Surgery → ECC ECC → Surgery → ECC/45 Gy	5-year OS: 41% 5-year OS: 41%	-	0.99	19
FNCLCC/FFCD	n = 113 n = 111	CF → Surgery (n = 113) Surgery (n = 111)	5-year OS: 38% 5-year OS: 24%	0.69 (0.50–0.95)	0.02	22
MAGIC	n = 250 n = 253	ECF → Surgery → ECF Surgery	5-year OS: 36% 5-year OS: 23%	0.75 (0.60–0.93)	0.009	20
MRC OEO-5	n = 446 n = 451	ECF → Surgery CF → Surgery	3-year OS: 39% 3-year OS: 42%	0.92 (0.79–1.08)	0.30	23
FLOT4	n = 360 n = 356	ECF → Surgery → ECF FLOT → Surgery → FLOT	3-year OS: 48% 3-year OS: 57%	0.77 (0.63–0.94)	0.012	25
ST03	n = 533 n = 530	ECF → Surgery → ECF ECF+Bev → Surgery → ECF+Bev	3-year OS: 50% 3-year OS: 48%	1.08 (0.91–1.29)	0.36	26

5-FU, 5-fluorouracil; Bev, bevacizumab; CF, cisplatin and 5-fluorouracil; CI, confidence interval; ECC, epirubicin, cisplatin, and capecitabine; ECF, epirubicin, cisplatin, and 5-fluorouracil; FLOT, docetaxel, oxaliplatin, leucovorin, and 5-fluorouracil; Gy, Gray; HR, hazard rate; OS, overall survival; XP, cisplatin and capecitabine.

**Table 2.** Targeted therapy trial for metastatic gastric adenocarcinoma.

Study	Number	Target	Treatment	Survival	HR (95% CI)	Reference
First-line setting						
ToGA	n = 298 n = 296	HER2	Trastuzumab + XP Placebo + XP	mOS: 13.8 months mOS: 11.1 months	0.74 (0.60–0.91)	34
TRIO-013/LOGiC	n = 249 n = 238	HER2/EGFR	Lapatinib + CapeOx Placebo + CapeOx	mOS: 12.2 months mOS: 10.5 months	0.91 (0.73–1.12)	59
EXPAND	n = 455 n = 449	EGFR	Cetuximab + XP Placebo + XP	mOS: 9.4 months mOS: 10.7 months	1.00 (0.87–1.17)	61
REAL3	n = 278 n = 275	EGFR	Panitumumab + EOC Placebo + EOC	mOS: 8.8 months mOS: 11.3 months	1.37 (1.07–1.76)	62
AVAGAST	n = 387 n = 387	VEGF	Bevacizumab + FP Placebo + FP	mOS: 12.1 months mOS: 10.1 months	0.87 (0.73–1.03)	64
RAINFALL	n = 326 n = 319	VEGFR	Ramucirumab + Cape/Cis Placebo + Cape/Cis	mOS: 11.2 months mOS: 10.7 months	0.68 (0.80–1.16)	63
RILOMET-1	n = 304 n = 305	MET	Rilotumumab + ECX Placebo + ECX	mOS: 8.8 months mOS: 10.7 months	1.34 (1.10–1.63)	65
METGastric	n = 279 n = 283	MET	Onartuzumab + FOLFOX Placebo + FOLFOX	mOS: 11.0 months mOS: 11.3 months	0.82 (0.59–1.15)	66
Cohen *et al*.	n = 60 n = 63	Hedgehog pathway	Vismodegib + FOLFOX Placebo + FOLFOX6	mOS: 11.5 months mOS: 14.9 months	-	74
FAST	n =84 n =77	Claudin18.2	IMAB362 + EOX Placebo + EOX	mOS: 13.2 months mOS: 8.4 months	0.51 (0.36–0.73)	78
Beyond second-line setting						
REGARD	n = 238 n = 117	VEGFR	Ramucirumab Placebo	mOS: 5.2 months mOS: 3.8 months	0.78 (0.60–0.99)	46
RAINBOW	n = 330 n = 335	VEGFR	Ramucirumab + paclitaxel Placebo + paclitaxel	mOS: 9.6 months mOS: 7.4 months	0.81 (0.68–0.96)	45
GATSBY	n = 228 n = 117	HER2	Trastuzumab emtansine Taxane	mOS: 7.9 months mOS: 8.6 months	1.15 (0.87–1.51)	58
TyTAN	n = 132 n = 129	HER2/EGFR	Lapatinib + paclitaxel Placebo + paclitaxel	mOS: 11.0 months mOS: 8.9 months	0.84 (0.64–1.11)	60
GRANITE-1	n = 439 n = 217	mTOR	Everolimus Placebo	mOS: 5.9 months mOS: 4.3 months	0.90 (0.75–1.08)	67
RADPAC	n = 150 n = 150	mTOR	Everolimus + paclitaxel Placebo + paclitaxel	mOS: 6.1 months mOS: 5.1 months	0.92 -	68
SHINE	n = 41 n = 30	FGFR	AZD4547 paclitaxel	mOS: 5.5 months mOS: 6.6 months	1.31 (0.89–1.95) ^a^	69
GOLD	n = 263 n = 262	PARP	Olaparib + paclitaxel Placebo + paclitaxel	mOS: 8.8 months mOS: 6.9 months	0.79 (0.63–1.00)	72

^a^80% confidence interval. Cape/Cis, capecitabine and cisplatin; CapeOx, capecitabine and oxaliplatin; CI, confidence interval; ECX, epirubicin, cisplatin, and capecitabine; EGFR, epidermal growth factor receptor; EOC/EOX, epirubicin, oxaliplatin, and capecitabine; FGFR, fibroblast growth factor receptor; FOLFOX, leucovorin, 5-fluorouracil, and oxaliplatin; FP, cisplatin and 5-fluorouracil; HER2, human epidermal growth factor receptor 2; HR, hazard rate; MET, mesenchymal–epithelial transition; mOS, median overall survival; mTOR, mammalian target of rapamycin; PARP, poly (ADP-ribose) polymerase; VEGF, vascular endothelial growth factor; VEGFR, vascular endothelial growth factor receptor; XP, cisplatin and capecitabine.
